# Changing Patterns of *Salmonella enterica* Serovar Rissen From Humans, Food Animals, and Animal-Derived Foods in China, 1995–2019

**DOI:** 10.3389/fmicb.2021.702909

**Published:** 2021-07-29

**Authors:** Mohammed Elbediwi, Daiwei Shi, Silpak Biswas, Xuebin Xu, Min Yue

**Affiliations:** ^1^Department of Veterinary Medicine, Institute of Preventive Veterinary Sciences, Zhejiang University College of Animal Sciences, Hangzhou, China; ^2^Agriculture Research Center, Animal Health Research Institute, Cairo, Egypt; ^3^National Institutes for Food and Drug Control, Beijing, China; ^4^Department of Microbiology Laboratory, Shanghai Municipal Center for Disease Control and Prevention, Shanghai, China; ^5^Hainan Institute of Zhejiang University, Sanya, China; ^6^State Key Laboratory for Diagnosis and Treatment of Infectious Diseases, National Clinical Research Center for Infectious Diseases, National Medical Center for Infectious Diseases, College of Medicine, The First Affiliated Hospital, Zhejiang University, Hangzhou, China; ^7^Zhejiang Provincial Key Laboratory of Preventive Veterinary Medicine, Hangzhou, China

**Keywords:** *Salmonella*, serovar Rissen, emerging serovar, public health, foodborne disease

## Abstract

Salmonellosis represents a growing threat to global public health. *Salmonella enterica* remains the leading cause of bacterial foodborne diseases in China. *Salmonella enterica* serovar Rissen (*S*. Rissen) has been recognized as one of the emerging serovars among humans in different countries worldwide. However, knowledge on the prevalence of *S*. Rissen in China is largely lacking. To address essential epidemiological information for *S*. Rissen in China, a total of 1,182 *S*. Rissen isolates recovered from samples across the food chain were collected from 16 provinces or province-level cities between 1995 and 2019. Risk factors due to the consumption of animal-derived food products were also analyzed. We found *S.* Rissen is widely distributed, especially in the Eastern and Southern parts of China, and there is an increasing frequency in recent years as evidenced by the greater number of isolates recovered in 2016, 2017, and 2018. Interestingly, the majority of *S.* Rissen isolates recovered in this study were from human samples (63.4%; 749/1182), remarkably, 58.4% (438/749) were from asymptomatic carriers. We obtained most of the *S.* Rissen isolates from humans from Guangxi (59.5%; 446/749) and Shanghai (29.5%; 221/749). Among 302 human diarrheal isolates (40.3%; 302/749), we found 44.6% (139/311) of *S.* Rissen in children with diarrhea (age below 10 years old). This is of clinical significance as diarrhea is one of the crucial causes of child mortality globally and our findings here highlighted the importance of *Salmonella* infections in Chinese children. Additionally, *S*. Rissen isolates were also found to be associated with pork and poultry products in China. This study projected the most updated national-wide study of *S*. Rissen isolates obtained from different sources in China over the past two decades. Continued surveillance is warranted to further monitor this emerging serovar in China and elsewhere over the world.

## Introduction

*Salmonella* is one of the most common causes of human diarrheal diseases resulting in a significant morbidity and mortality burden over the world (Centers for Disease Control Prevention, 2013; Crump et al., 2015; Xu X. et al., 2020). It is also being considered as the leading cause of childhood diarrheal diseases in developing countries (Ince et al., 2012; Bula-Rudas et al., 2015) especially in China. *Salmonella* spp. are a group of bacteria that can survive in animals, humans, and the environment (Boyle et al., 2007; Velge et al., 2012; Elbediwi et al., 2020a). *Salmonella* is an important foodborne pathogen that causes gastroenteritis and bacteremia in humans (Hohmann, 2001; Boyle et al., 2007; Majowicz et al., 2010; Crump et al., 2015; Wang et al., 2019). Particularly, *Salmonella* infections by certain serovar are one of the significant causes for economic losses in the livestock production industry (Paudyal et al., 2018; Xu Y. et al., 2020).

Non-typhoidal *Salmonella* (NTS) is a common etiological agent of human diarrheal disease worldwide (Majowicz et al., 2010). Although many serovars have been identified, most of the human infections are generally caused by a limited number of *Salmonella* serovars. *Salmonella* enterica serovar Typhimurium and Enteritidis are among the most frequent *Salmonella* serovars which cause human salmonellosis, but several other serovars are being reported to be more prevalent in certain regions (Biswas et al., 2019; Jajere, 2019).

A bilateral changing trend in association between previously under-reported *Salmonella* serovars such as *Salmonella* Rissen and *Salmonella* Derby causing foodborne salmonellosis and increasing pork and poultry production has been observed (Padungtod and Kaneene, 2006; Jiang et al., 2021). *Salmonella enterica* serovar Rissen (*S*. Rissen) is a frequently reported serovar around different countries with a significant association with intensive pig industry.

*S*. Rissen is reported as the top three serovars in swine products in Southeast Asia and Europe (Schmidt et al., 2012). Several studies also detect *S*. Rissen in slaughtered pigs in some European Countries (Vieira-Pinto et al., 2006; Wales et al., 2009; Belsue et al., 2011; Arguello et al., 2013). The recent report published by European Food Safety Authority and European Centre for Disease Prevention and Control (EFSA and ECDC) identified *S*. Rissen among the twenty most common *Salmonella* serovars linked with human salmonellosis and as one of the top ten serovars associated with swine and poultry products in the European Union (European Food Safety Authority and European Centre for Disease Prevention and Control, 2015). *Salmonella* Rissen infections in humans have also been reported from several countries (Foley et al., 2005; Hendriksen et al., 2008; Higa, 2011). The risk of *Salmonella* infection in humans including the increase of multidrug resistance in *Salmonella* spp. highlights the necessity for the continuous surveillance of emerging *Salmonella* serovars, including Rissen (Biswas et al., 2019; Elbediwi et al., 2019).

To date, the knowledge on *Salmonella* Rissen epidemiological prevalence and disease burden in China is largely unknown. Therefore, to address these key knowledge gaps in *S.* Rissen infection in China, our study aimed to establish an epidemiological relationship of 1,182 *S*. Rissen isolates obtained from humans, food animals, food of animal origin, and environment over a period ranging from 1995 to June 2019 in China. We also investigated *S*. Rissen infection in children, aiming to understand the clinical epidemiology of *S*. Rissen isolates in Chinese children. Given the importance of NTS infection in worldwide foodborne illnesses and childhood diarrhea, knowledge of national-wide epidemiology for emerging NTS serovars could guide appropriate control measurements and policy planning. Updated information about the epidemiology and prevalence of different *Salmonella* serovars in specific areas may facilitate precision public health interventions for mitigation of emerging pathogens.

## Materials and Methods

### Bacterial Isolates

A total of 1,182 *S*. Rissen isolates were used in this study. *S*. Rissen isolates were obtained from a collection of (>30,000) isolates as a part of the Chinese Local Surveillance System. These isolates were collected from human samples (diarrhea, urine infections, bacteremia, and asymptomatic carriers), live animal samples (pigs, chicken), food samples (pork, poultry meat, poultry products, and seafood), and environmental samples (water and soil). They were originated from 16 provinces or province-level cities (Beijing, Chongqing, Fujian, Guangdong, Guangxi, Hebei, Henan, Hubei, Jiangsu, Shandong, Shanghai, Shanxi, *Shenzhen*, Sichuan, Xinjiang, and Zhejiang) in China. The meta-data for all *Salmonella* isolates can be found in Supplementary Table 1.

### Isolation and Characterization of Bacteria

Isolation of the microorganism was conducted based on the protocol recommended by the World Organization for Animal Health Terrestrial Manual (Elbediwi et al., 2020b, Elbediwi et al., 2021). Briefly, human (feces, blood, and urine), animal (feces), food or environmental samples were subjected into 10 mL pre-enrichment in buffered peptone water (Oxoid, United Kingdom). Following the initial pre-enrichment in buffered peptone water, 0.1 mL of the pre-enriched samples were added to 10 mL of Rappaport Vassiliadis broth (Oxoid, United Kingdom) and incubated at 42°C for 24 h. The enriched samples were streaked onto Xylose Lysine Desoxycholate (XLD) (Oxoid, United Kingdom). Plates were then incubated at 37°C for 18–24 h. Spherical transparent red or pink colonies with or without typical black centers on XLD, were selected as presumptive *Salmonella* colonies. The bacterial isolates were then confirmed using polymerase chain reaction (PCR). DNA extraction was done by boiling method. PCR for enterotoxin *stn* gene for the confirmation of the *Salmonella* was performed as recommended (Deguenon et al., 2019).

### *Salmonella* Serotyping

The pure colonies of bacteria were seeded in Luria-Bertani (LB) broth for serotyping. For further serotyping analysis, the PCR confirmed *Salmonella* isolates were performed by slide agglutination method to define O and H antigens using commercial antisera (SSI Diagnostica, Denmark), and the results were interpreted according to the White-Kauffmann-Le Minor scheme (Grimont and Weill, 2007).

### Statistical Analysis

The chi-square test variances were used to test the significant differences in the prevalence of *Salmonella* isolates between samples collected from different geographical regions, sampling origins, human sex, human age groups, in addition to the difference between the prevalence of asymptomatic carriers and diseased humans if information is available. *P-*values less than 0.05 were considered statistically significant. Statistical analysis of the results was performed with GraphPad Prism 7.

## Results

### Emergence and Geographical Distribution of *S*. Rissen Serovar in China

**FIGURE 1:**
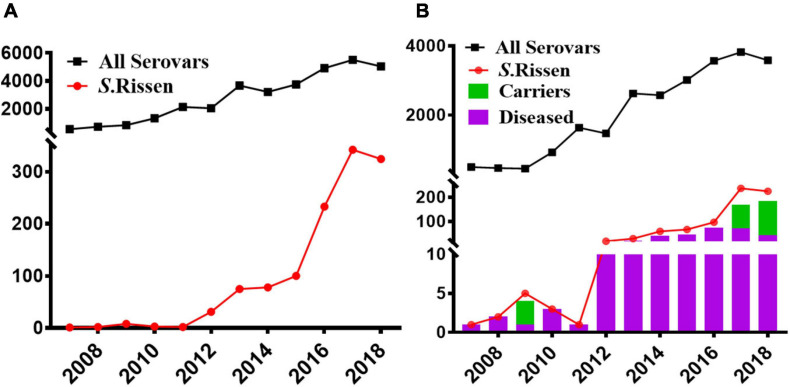
Increasing trend of the *Salmonella* serovar Rissen in China between 1995 and June 2019. **(A)** An emerging trend of *S*. Rissen in China as compared to other *Salmonella* serovars. **(B)** An emerging trend of *S*. Rissen serovar in humans in addition to the prevalence of clinical patients and asyndromatic carriers in China as compared to other *Salmonella* serovars.

**FIGURE 2:**
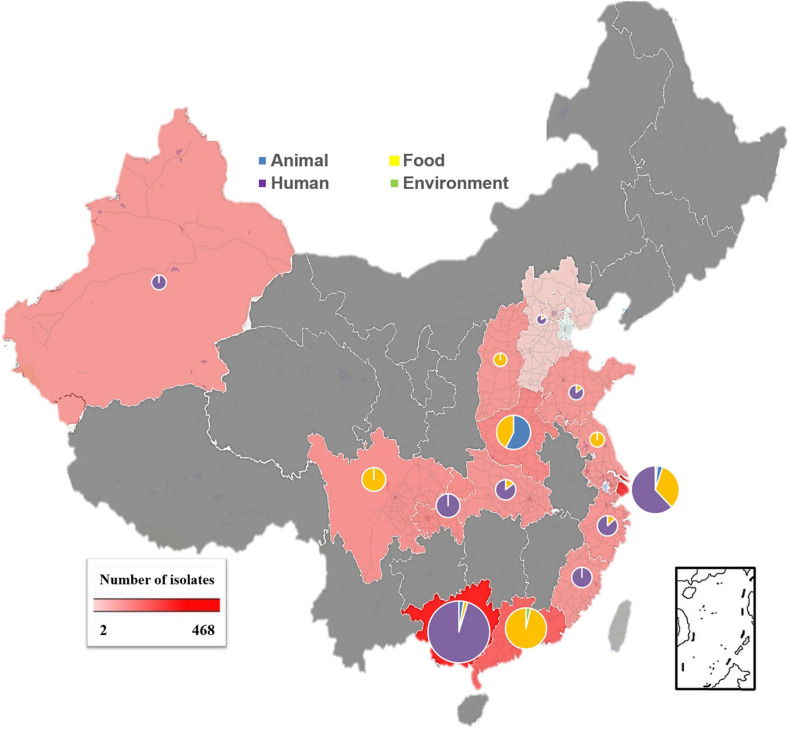
Geographical distribution of *S*. Rissen isolates obtained from four established sources (humans, foods of animal origin, food animals, and environment) in China during 1995–2019. Pie charts reveal that the different sources for samples in each province and the size of charts is according to the actual number of the isolates obtained from each province. Blue color for animal samples, yellow color for food samples, green color for environmental samples and violet color human samples.

**FIGURE 3:**
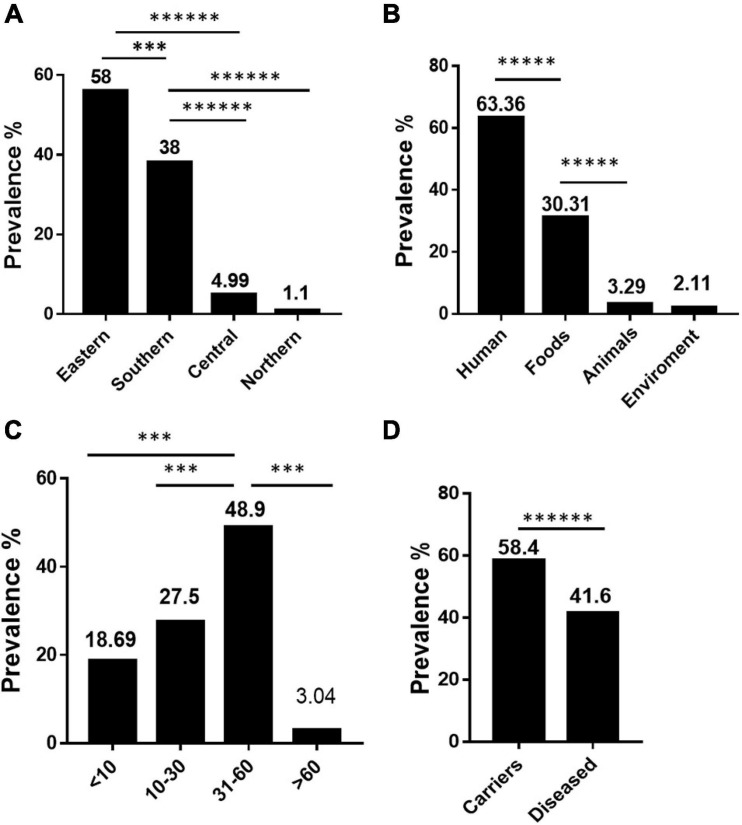
The prevalence and dynamics among *S.* Rissen isolates examined in this study. **(A)** Difference in prevalence of *S.* Rissen isolates collected from different geographical parts. **(B)** Difference in prevalence of *S.* Rissen isolates collected from different origins. **(C)** Difference in prevalence of *S.* Rissen isolates collected from different human age groups. **(D)** Difference in prevalence of *S.* Rissen between the asymptomatic carriers and diseased patients. *P-*values less than 0.05 were considered statistically significant. *Y*-axis revealed to the prevalence %. The threshold of significance for the *P*-values indicated as follows: ^*⁣*⁣*⁣*⁣**^P < 0.0001; ^*⁣*⁣*⁣**^P < 0.0001; ^∗∗∗^*P* < 0.001.

Our data showed that *S*. Rissen prevalence was increasingly detected in recent years in China as evidenced by the greater number of isolates recovered in 2016, 2017, and 2018 (Figures 1A,B). Furthermore, our data suggest that *S*. Rissen is an emerging serovar in China. The prevalence of *S*. Rissen in this study is of concern as evidenced by the distribution of the serotype in 16 provinces or province-level cities consisting of almost all geographical regions in China. The *S*. Rissen isolates were mostly obtained from the southern (51.1%; 604/1182) and eastern (37.9%; 448/1182) parts of China (Figure 2). Notably, *S*. Rissen was more prevalent in Guangxi 39.59% (468/1182) and Shanghai 30.11% (356/1182), which was also indicated a region-specific distribution (Figure 2). Statistical analysis based on the chi-square test variances showed that there is a significant difference between the prevalence of *Salmonella* isolates collected from the Southern part and those collected from Central and Northern parts (*P* < 0.00001), and between the isolates collected from the Southern part and Eastern part (*P* < 0.001) (Figure 3A), and also showed no significant difference between the different provinces (*P* > 0.05) (data not shown).

Our results also showed that the majority of *S.* Rissen isolates studied in this study were obtained from humans (63.36%; 749/1182) followed by foods (31.1%; 368/1182), animals (3.29%; 39/1182), environment (2.11%, 25/1182), and also displayed statistically significant difference between the isolates recovered from the food samples and those collected from human and animal samples (*P* < 0.0001) (Figure 3B). To the best of our knowledge, this is the first study to establish an epidemiological relationship of 1,182 *S*. Rissen isolates obtained from humans, animals, and food products over a period of two decades in China.

### Prevalence of *S.* Rissen Isolates in Human Samples

Our results showed that the majority of *S.* Rissen isolates studied in this study were obtained from humans (63.36%) in China which is of great clinical significance. However, most of the *S*. Rissen isolates causing human salmonellosis were from Shanghai 29.5% (221/749) (Figure 2). Some isolates derived from cases with diarrhea were also obtained from Chongqing and Zhejiang. Importantly, we also noticed an accumulation in the number of isolates from the asymptomatic carriers in 2017, and 2018 (Figure 1B), majority of these isolates are located and prevalent in Guangxi, Southern China. Additionally, 54% (407/749) of *S*. Rissen isolates were obtained from females. Out of 407, 134 isolates were recovered from diseased females. On the other hand, 46% (342/749) of males were affected with *S*. Rissen isolates. Out of these, 51.75% (177/342) of males showed disease syndromes caused by *S*. Rissen. We also noticed that there was no statistically significant difference between the isolates obtained from males and females in this study (*P* > 0.05) (data not shown).

Interestingly, most of the *S*. Rissen isolates from humans were recovered from Guangxi (62.5%; 468/749) and Shanghai (47.5%; 356/749) from different age-group (Figure 3C). It was noteworthy that the distribution of *S*. Rissen in different age-group was not even. 18.69% (140/749) *S*. Rissen isolates were found in the age group under 10; 27.50% (206/749) isolates were found in the age group between 10 and 30, 48.99% (367/749) isolates were found in the age group between 31 and 60, and 3.04% (36/749) isolates were found in the age group above 60 (Figure 3C). Statistical analysis showed that the prevalence of *Salmonella* isolates collected from human samples in the age group between 31 and 60 was statistically different from those collected from other age groups (*P* < 0.001) (Figure 3C). Our study also revealed that 42% (311/749) and 58% (438/749) of *S*. Rissen isolates from diseased (diarrheal 40.8% (302/749) and bacteremia and urine infections 1.2% (9/749) and asymptomatic carriers, respectively, with a significant difference (*P* < 0.000001) (Figure 3D).

### Prevalence of *S*. Rissen Isolates in Live Animals, Food of Animal Origin, and Environmental Samples

Among isolates obtained from live animals and food products, we found that sample obtained from live pigs (84.61%, 33/39) and pork products (65.56%, 253/386) were the highest prevalent with *S*. Rissen isolates followed by live chicken (15.39%, 6/39) and chicken meat (22.53%, 87/386), respectively (Supplementary Table 1).

Furthermore, the highest prevalence of serovar Rissen in the pig and chicken production chain was observed in Guangdong, followed by Shanghai and Henan provinces (Supplementary Table 1). There was no statistically significant difference between the isolates obtained from food of different sources in this study (*P* > 0.05) (data not shown). We also noticed that only 3% of S. Rissen isolates were obtained from seafood including (different types of mollusks, including razor clam, snail, oyster) and only one isolate from minced fish from Shanghai and 2% from beef (Supplementary Figure 1). Our results also showed that (2.11%, 25/1182) isolates were obtained from environmental sources. Two isolates were obtained from soil and the other 23 isolates were obtained from water samples.

## Discussion

**FIGURE 4:**
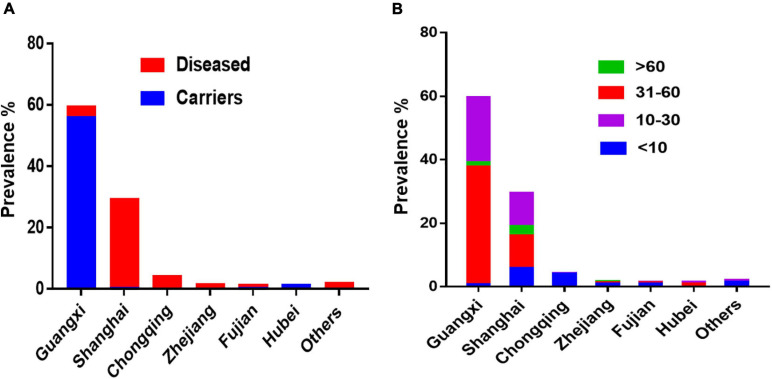
The prevalence of *S*. Rissen human isolates in China. **(A)** Prevalence of *S.* Rissen isolates from clinics and asymptomatic carriers in different provinces or province-level cities in China. **(B)** Prevalence of *S*. Rissen isolates from different age-group of humans recovered from different provinces or province-level cities in China. This suggests that the most prevalent age-group was the children under the age of 10.

*S.* Rissen is one of the most common serovars found in gastrointestinal patients, swine herds, pork, and chicken products in different parts of the world (Angkititrakul et al., 2005; Vo et al., 2006; Kumar et al., 2009; Lim et al., 2009). In this study, we highlighted that *S*. Rissen is an emerging serovar in China, which has been increasingly detected in recent years in almost all geographical regions in China. The first case of *S.* Rissen was described in two immunosuppressed children from turtles in France in 1990 (Mallaret et al., 1990). *S.* Rissen isolates are also obtained from patients with diarrhea during the period from 1985 to 1994 in Yamanashi Prefecture, Japan (Kaneko, 1995). Following these two reports, serovar Rissen was reported from many other countries (Oliveira et al., 2002; Song et al., 2005; Vaeteewootacharn et al., 2005; Inthavong et al., 2006; Riaño et al., 2006; Vieira-Pinto et al., 2006; Vo et al., 2006; Hendriksen et al., 2008; Kumar et al., 2009; Wales et al., 2009). In these countries, *S*. Rissen was associated with either animal or animal-derived foods. However, in this study the majority of *S.* Rissen isolates derived from humans (63.4%; 749/1182). This is of significant clinical importance concerning the emerging trend of this serovar. Additionally, all isolates obtained from children under the age of 10 years were isolated from clinical cases of diarrhea. Diarrhea remains a significant cause of morbidity and mortality among children globally (Walker et al., 2013). Compared with the surveillance data, we found that the prevalence of *S.* Rissen in diarrheal children was much higher in Shanghai (Figure 4). A recent hospital-based case-control study reported that the prevalence of NTS in diarrheal children was 9.3% in Shanghai (Chang et al., 2017). Li Y. et al. (2014) also reported an increasing NTS infection in pediatric cases with acute gastroenteritis. Another study showed that 34% of NTS diarrheal cases occurred in children under 5-years old in China (Ran et al., 2011). Younger children are likely more vulnerable by diarrhea-causing pathogens because of their food habits as they have different exposure pathways than those of adults due to their immunological condition and developmental stage. For example, young children involve in normal exploratory behaviors including hand-to-mouth and object-to-mouth behaviors, and non-nutritive ingestion which may increase exposure over that in adults. The amount of food that children consume per kilogram of body weight is higher than that of the adult because children not only need to maintain homeostasis, as adults do, but are growing. If the food or liquid contains a contaminant, children may receive more of it relative to their size than adults. In addition, children consume a specific type of food (American Academy of Pediatrics Committee on Environmental Health, 2003; Mahoney and Moy, 2005). The ingestion of contaminated food, mainly foods of animal origin, is recognized as the most possible source of NTS transmission to humans, with a huge worldwide impact on human health (Crump et al., 2015; Arya et al., 2017).

Our results also showed that 58% of *S*. Rissen isolates were isolated from asymptomatic carriers and may be due to the number of ingested bacteria since symptoms of bacterial infection with NTS depend on the number of ingested bacteria. In general, the number of bacteria that could cause disease symptoms for a healthy human host ranges from 10^6^ to 10^8^ organisms (Xu et al., 2010; Gut et al., 2018). Carriage of *Salmonella* and other pathogens can be temporary or chronic. Several studies have reported different periods of asymptomatic shedding from 3 months (Copyright and License information, 1910; Vogelsang and Bøe, 1948) to 12 months (Musher and Rubenstein, 1973; Pires et al., 2014). Unlike, *S.* Typhi, lifelong persistence of NTS was not detected, and 8 years was the maximal recognized time of NTS carriage (Yue, 2016). The majority of the persistent infection in patients were immunocompromised. Smaller doses of the ingested pathogen can produce diseases in high−risk groups which might be the reason for higher rates of NTS infection in children, especially those under 5 years of age, and immunocompromised patients (Bula-Rudas et al., 2015). On the other hand, a higher dose of ingested bacteria correlates with a more severe disease. In some cases, clinical symptoms of bacterial infection may be mild or the person may be asymptomatic; and sometimes because of this, the data about the true incidence of infection are underestimated (Bula-Rudas et al., 2015; Paudyal et al., 2020).

This study also showed that *Salmonella* serovar Rissen isolates were widespread in live animals and foods from retail markets in different regions or parts of China. *S*. Rissen is frequently associated with pigs and pig products as reported previously (Musher and Rubenstein, 1973; Jiang et al., 2019; Paudyal et al., 2020). Recently, 24.1% of *S*. Rissen isolates were detected from pork samples in Guangdong province, China (Lertworapreecha et al., 2013; Boonkhot et al., 2015). Recently, 24.1% of *S*. Rissen isolates were detected from pork samples in Guangdong province, China (Zhang et al., 2018). Similar finding was reported previously by Hendriksen et al. (2008) in Denmark. *S.* Rissen is described to be among the most common serovars found in Thailand pig industries (Dorn-In et al., 2009) and has been shown to efficiently transmit from pigs to humans along the food chain (Sanguankiat et al., 2010; Prasertsee et al., 2019). Other studies reported *S*. Rissen (17.1%) found in piglets in Spain (Casanova-Higes et al., 2019); and accounted for 10.5% of *Salmonella* recovered from French slaughterhouse (Bridier et al., 2019); 31.25% of *S*. Rissen were identified on pig carcasses in Italy (Bonardi et al., 2016); 57.1% of *S*. Rissen from pigs were found from Shandong province, China in 2017 (Zhao et al., 2017). The high levels of *Salmonella* Rissen contamination suggest Hazard Analysis and Critical Control Point (HACCP) system for the pork being sold in retail outlets in many countries should be improved or adjusted. Therefore, the consumption of contaminated swine products is considered one of the most important sources of human infection resulting in *Salmonella* outbreaks. The prevalence of *S*. Rissen in pork is of concern because it has been responsible for increasing sporadic human cases in China.

Additionally, food of animal origins such as poultry, its products, and eggs are usually associated with human salmonellosis (European Food Safety Authority and European Centre for Disease Prevention and Control, 2015; World Health Organization, 2015; Liu et al., 2020). Poultry products are a vital source of *Salmonella* in the United States (Andino et al., 2014; Barbour et al., 2015) and Europe (European Food Safety Authority and European Centre for Disease Prevention and Control, 2015). Other study from Thailand also found *S.* Rissen among the most common serovars in chicken meat (Padungtod and Kaneene, 2006). The presence of *Salmonella* in retail meat and its related products have often led them to be unsafe for human consumption (Centers for Disease Control Prevention, 2013). Our result also highlights the importance of the chicken reservoir as an alternative source of *S.* Rissen infection to humans. The egg-related *Salmonella* outbreaks have decreased over time due to the use of antibiotics in the poultry industry and more strict preventive measures as evidenced by this study as we found only two *S*. Rissen isolates from eggs in the whole study (Supplementary Table 1).

We also noticed that *S*. Rissen isolates were detected in seafood and minced fish in our study. Isolation of *Salmonella* serovars from fish, live molluscan shellfish from the marine environment has been reported previously in Cambodia (Nadimpalli et al., 2019) and Spain (Martinez-Urtaza et al., 2003). A recent report verified that three *S*. Rissen isolates were recovered from ready-to-eat mussels between 2012 and 2016 in northwest Spain (Lozano-Leon et al., 2019), and another report from the seafood in India (Kumar et al., 2009). It is important to know that *Salmonella enterica*, including serovar Rissen, can be transmitted worldwide by international travel and food trade (Hendriksen et al., 2008). A previous study reported that consumption of local and imported swine products and travel history from Thailand were risk factors for *S*. Rissen infection in Danish patients in Denmark (Hendriksen et al., 2008).

Indeed, contamination by *Salmonella* in animal-derived foods in China is a serious issue, posing increasing the risk for human infections. The presence of *S*. Rissen in different foodstuffs highlights the need for continuing surveillance of these food products. Our results suggest that animal-derived foods should be paid more attention to mitigate the dissemination of *Salmonella*. These findings highlight the importance of strict prevention and control measures in the pork and poultry production process to ensure food safety along the food chain in China.

## Conclusion

This study presented the most comprehensive and updated epidemiological description of emerging *S*. Rissen in humans, animals, and animal foods in China. Here, original data on *Salmonella* prevalence and associated microbial ecology were collected and the dynamics of *S.* Rissen infection have been extensively studied. This investigation may have potential benefits for future *S.* Rissen surveillance and outbreak detection in China. The updated knowledge may lead to a better understanding of the prevalence and disease burden caused by *S*. Rissen in China and in other countries. This information will provide support for the development of novel approaches to mitigate *Salmonella* infections along the food production chain and in humans. *Salmonella* control strategies from farm to table should focus on all stages of the food production chain to reduce contamination levels and consumer risk. Moreover, more research regarding the characteristics of the dissemination of *S*. Rissen in China is highly needed and continued surveillance of this serovar is necessary as it can cause human diseases as well as asymptomatic carrier, which may represent as the reservoir for human transmissions. This study provides a framework for understanding *Salmonella* epidemiology from a national-wide to a global perspective and these findings here may offer valuable information for developing future *Salmonella* surveillance systems globally.

## Data Availability Statement

The original contributions presented in the study are included in the article/Supplementary Material, further inquiries can be directed to the corresponding author.

## Ethics Statement

The studies involving human participants were reviewed and approved by the Chinese National CDC. The patients/participants provided their written informed consent to participate in this study.

## Author Contributions

ME analyzed the data and finalized the figures. ME and SB wrote the manuscript. DS and XX did the experiment and collect the data. MY conceived the idea and assisted with data analysis and writing. All authors read, revised, and approved the final manuscript.

## Conflict of Interest

The authors declare that the research was conducted in the absence of any commercial or financial relationships that could be construed as a potential conflict of interest.

## Publisher’s Note

All claims expressed in this article are solely those of the authors and do not necessarily represent those of their affiliated organizations, or those of the publisher, the editors and the reviewers. Any product that may be evaluated in this article, or claim that may be made by its manufacturer, is not guaranteed or endorsed by the publisher.

## Supplementary Material

Supplementary Figure 1 Pie chart of the prevalence of *S*. Rissen isolates obtained from food of animal-origin samples in China.
