# Supplementary figures and images for: Changing Patterns of Salmonella enterica Serovar Rissen From Humans, Food Animals, and Animal-Derived Foods in China, 1995–2019
Source: Front Microbiol. 2021 Jul 29;12:702909. doi: 10.3389/fmicb.2021.702909 (PMC8358327; doi:10.3389/fmicb.2021.702909)

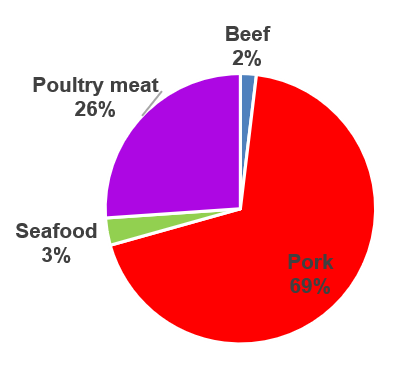

Supplement: Supplementary Figure 1 — Pie chart of the prevalence of S. Rissen isolates obtained from food of animal-origin samples in China. [file Image_1.TIF]
